# TGF-β1-induced differentiation of SHED into functional smooth muscle cells

**DOI:** 10.1186/s13287-016-0459-0

**Published:** 2017-01-23

**Authors:** Jian Guang Xu, Shao Yue Zhu, Boon Chin Heng, Waruna Lakmal Dissanayaka, Cheng Fei Zhang

**Affiliations:** 1 Comprehensive Dental Care, Endodontics, Faculty of Dentistry, The University of Hong Kong, Pokfulam, Hong Kong China; 2 HKU Shenzhen Institute of Research and Innovation, Hong Kong, China

**Keywords:** Tissue engineering, Angiogenesis, Stemness, Dental pulp stem cells, Smooth muscle cells

## Abstract

**Background:**

Adequate vascularization is crucial for supplying nutrition and discharging metabolic waste in freshly transplanted tissue-engineered constructs. Obtaining the appropriate building blocks for vascular tissue engineering (i.e. endothelial and mural cells) is a challenging task for tissue neovascularization. Hence, we investigated whether stem cells from human exfoliated deciduous teeth (SHED) could be induced to differentiate into functional vascular smooth muscle cells (vSMCs).

**Methods:**

We utilized two cytokines of the TGF-β family, transforming growth factor beta 1 (TGF-β1) and bone morphogenetic protein 4 (BMP4), to induce SHED differentiation into SMCs. Quantitative real-time polymerase chain reaction (RT-qPCR) was used to assess mRNA expression, and protein expression was analyzed using flow cytometry, western blot and immunostaining. Additionally, to examine whether these SHED-derived SMCs possess the same function as primary SMCs, in vitro Matrigel angiogenesis assay, fibrin gel bead assay, and functional contraction study were used here.

**Results:**

By analyzing the expression of specific markers of SMCs (α-SMA, SM22α, Calponin, and SM-MHC), we confirmed that TGF-β1, and not BMP4, could induce SHED differentiation into SMCs. The differentiation efficiency was relatively high (α-SMA^+^ 86.1%, SM22α^+^ 93.9%, Calponin^+^ 56.8%, and SM-MHC^+^ 88.2%) as assessed by flow cytometry. In vitro Matrigel angiogenesis assay showed that the vascular structures generated by SHED-derived SMCs and human umbilical vein endothelial cells (HUVECs) were comparable to primary SMCs and HUVECs in terms of vessel stability. Fibrin gel bead assay showed that SHED-derived SMCs had a stronger capacity for promoting vessel formation compared with primary SMCs. Further analyses of protein expression in fibrin gel showed that cultures containing SHED-derived SMCs exhibited higher expression levels of Fibronectin than the primary SMCs group. Additionally, it was also confirmed that SHED-derived SMCs exhibited functional contractility. When SB-431542, a specific inhibitor of ALK5 was administered, TGF-β1 stimulation could not induce SHED into SMCs, indicating that the differentiation of SHED into SMCs is somehow related to the TGF-β1-ALK5 signaling pathway.

**Conclusions:**

SHED could be successfully induced into functional SMCs for vascular tissue engineering, and this course could be regulated through the ALK5 signaling pathway. Hence, SHED appear to be a promising candidate cell type for vascular tissue engineering.

**Electronic supplementary material:**

The online version of this article (doi:10.1186/s13287-016-0459-0) contains supplementary material, which is available to authorized users.

## Background

Formation of blood vessels involves endothelial cells lining the interior surface of the tubule wall, and perivascular cells (vSMCs and pericytes) covering the exterior of the vascular tube. Through direct interaction with endothelial cells, perivascular cells play key roles as guiders that facilitate sprouting of endothelial cells during generation of new capillaries from immature blood vessels [1], as well as in regulating both the stability and contractile function of mature blood vessels [2]. Therefore, generating functional perivascular cells is crucial for vascular tissue engineering.

Most studies on vascular tissue engineering utilize commercially available primary cells, such as human umbilical vein endothelial cells (HUVECs), human umbilical artery endothelial cells (HUAECs), vascular smooth muscle cells (vSMCs), and pericytes (PCs) [3, 4]. Nevertheless, the scarce availability of human tissue sources for primary cell isolation, limited proliferative capacity, slow expansion rate, and variability of tissue-specific phenotype would hinder the clinical translation of mature ECs and vSMCs [4]. Therefore, inducing human embryonic, induced pluripotent, or adult stem cells, and endothelial progenitor cells to generate ECs and vSMCs have been explored [5, 6]. However, tumorigenicity, immunocompatibility and lack of precise control of differentiation are challenges that need to be addressed.

Dental pulp stem cells can be readily harvested from exfoliated deciduous or wisdom teeth, without imposing additional discomfort or injury on patient donors. Notably, stem cells from human exfoliated deciduous tooth (SHED) exhibit faster and more extensive proliferative capacity than bone marrow-derived mesenchymal stem cells (BMMSCs) [7], and can differentiate into functional endothelial-like cells [8, 9]. Currently within the scientific literature, there is a lack of data on derivation of vSMCs from SHED, and the utilization of these cells to generate functional blood vessels.

Transforming growth factor beta 1 (TGF-β1) proteins are the most potent soluble growth factors commonly utilized for inducing stem/progenitor cell differentiation into SMCs. The TGF-/Smad signaling pathway is critical for differentiation of multipotent mesenchymal progenitors into SMCs [10]. TGF-β stimulation and Notch activation have been reported to induce differentiation of mesenchymal stem cells (MSCs) and human embryonic stem cells (hESCs) into SMCs [11]. Vo et al. [12] utilized TGF-β combined with platelet-derived growth factor (PDGF-BB) to induce differentiation of hESCs into smooth muscle cells. Additionally, TGF-β1 alone or in combination with other factors can also effectively induce adult stem cells into the SMC lineage. For example, adipose-derived stem cells exposed to a combination of TGF-β1 and bone morphogenetic protein 4 (BMP4), could differentiate into SMCs [13]. TGF-β1 combined with ascorbic acid was demonstrated to induce bone marrow-derived mesenchymal stem cells into SMCs [14].

Hence, the objective of this study was to differentiate SHED into SMCs by TGF-β1 alone or in combination with BMP4, and to investigate the functionality of the derived SMCs.

## Methods

### Cell culture

SHED were purchased from ALLCELLS (Alameda, CA, USA), and were evaluated for their “stemness” before induction. The expression of stem cell associated phenotypic markers, CD73, CD90, CD105, and CD45, were analyzed by flow cytometry (Additional file 1: Figure S1). In addition, the multiple differentiation capacity of SHED was confirmed using adipogenic, osteogenic, and neurogenic induction media (Additional file 2: Figure S2).

HUVECs and primary SMCs were purchased from ScienCell (Carlsbad, CA, USA) and were cultured in fully supplemented endothelial growth medium (EGM-2, Lonza, Walkersville, MD, USA) and smooth muscle cell medium (SMCM, ScienCell) respectively.

### In vitro differentiation and expansion of SHED-derived SMCs

SHED were plated at a density of 3 × 10^3^ cells/cm^2^ and cultured in α-MEM supplemented with 10% (v/v) FBS until 70% confluency. Differentiation was induced in α-MEM with 5% (v/v) FBS supplemented with TGF-β1 (2.5, 5, 10, 20, 30 ng/ml) (Peprotech, Rocky Hill, NJ, USA) and/or BMP4 (5, 10, 30 ng/ml) (Peprotech), with medium being refreshed every other day. On day 7, the induced SHED were expanded in α-MEM with 5% (v/v) FBS supplemented with 1 ng/ml TGF-β1, to obtain SHED-derived SMCs at passage 1 to 2.

### RT-qPCR

Real-time quantitative PCR was performed according to previously described protocol [15]. The utilized primer sequences were as follows: GAPDH, 5′-TGCACCACCAACTGCTTAGC-3′, 5′-GGCATGGACTGTGGTCATGAG-3′; α-SMA, 5′-CCGACCGAATGCAGAAGGA-3′, 5′-ACAGAGTATTTGCGCTCCGAA-3′; SM22α, 5′-AGTGCAGTCCAAAATCGAGAAG-3′, 5′-CTTGCTCAGAATCACGCCAT-3′; Calponin,5′-GTCAACCCAAAATTGGCACCA-3′, 5′-ACCTTGTTTCCTTTCGTCTTCG-3′.

### Western blot

Western blot analyses of protein expression were also performed as previously described [15]. The following primary antibodies were used: mouse monoclonal anti-β-actin monoclonal antibody (sc-47778; Santa Cruz Technology, Dallas, TX, USA); rabbit polyclonal anti-SM22 alpha antibody (ab14106; Abcam, Cambridge, MA, USA); rabbit monoclonal anti-Calponin antibody (ab46794; Abcam); mouse monoclonal anti-Fibronectin antibody (MA5-11981; Thermo Fisher Scientific, Waltham, MA, USA); and Smad2/3 antibody kit (#12747; Cell Signaling Technology, Danvers, MA, USA).

### Immunocytochemistry

After differentiation, SHED-derived SMCs were fixed with 4% (w/v) cold PFA for 15 minutes and washed with PBS. The cells were then permeabilized using 0.1% (v/v) Triton-X100 for 10 minutes. Primary antibodies against α-SMA (A2547; Sigma-Aldrich, St. Louis, MO, USA), SM22 alpha (ab14106; Abcam), smooth muscle Myosin heavy chain 11 (ab683; Abcam) and Calponin (ab46794; Abcam) were utilized for immunocytochemical staining. Goat anti-mouse immunoglobulin G (IgG) H&L (Alexa Fluor® 488) pre-adsorbed (ab150117; Abcam) and goat anti-rabbit IgG H&L (Alexa Fluor® 488) (ab150077; Abcam) were utilized as secondary antibodies. Primary SHED and SMCs were used as negative and positive controls. The images were viewed and captured under fluorescence microscopy.

### Flow cytometry analysis

BD Cytofix/Cytoperm™ Fixation/Permeabilization Kit (554714; BD Biosciences, San Jose, CA, USA) was used in flow cytometry to detect the expression of the various aforementioned smooth muscle cell markers by SHED-derived SMCs. Cell dissociation solution (C5914; Sigma-Aldrich) was used to disperse the SHED-derived SMCs. After fixation and permeabilization, cells were incubated for 2 hours with primary antibodies against α-SMA (ab124964; Abcam), SM22 alpha (ab14106; Abcam), smooth muscle Myosin heavy chain 11 (ab683; Abcam), Calponin (ab46794; Abcam), and then washed with PBS. This was followed by incubation with the appropriate secondary antibodies: goat anti-mouse IgG H&L (Alexa Fluor® 488) pre-adsorbed (ab150117; Abcam) and goat anti-rabbit IgG H&L (Alexa Fluor® 488) (ab150077; Abcam). In this study, mouse IgG1, kappa monoclonal (ab170190; Abcam) and rabbit IgG, monoclonal [EPR25A] (ab172730; Abcam) were utilized as the isotype control. Flow cytometry data was analyzed with FACSVerse software (BD Biosciences).

### In vitro Matrigel angiogenesis assay

To investigate whether SHED-derived SMCs could promote the stability of blood vessel structures, the in vitro Matrigel angiogenesis assay was performed according to established protocols [16], where the time course of vascular structure was evaluated to assess its stability. Briefly, HUVECs were seeded at 36,000 cells per well of 48-well plates that had been pre-coated with Growth Factor-Reduced Matrigel (354230, BD Biosciences). After 6 hours incubation at 37 °C and 5% CO_2_, different groups of cells (group 1: SHED-derived SMCs; group 2: SHED; group 3: primary SMCs) were added to Matrigel surface at a density of 7200 cells/well. Images were captured over 48 hours. For some applications, CellTracker® fluorescent probes (Life Technologies, Carlsbad, CA, USA) were used to label different cell types to distinguish them within vessel-like structures. HUVECs were stained with red fluorescence (Cell Tracker® Red CMTPX Dye, Life Technologies), while SHED-derived SMCs/primary SMCs were stained with green fluorescence (Cell Tracker® Green CMFDA Dye, Life Technologies). Images were captured under fluorescence microscopy, and were analyzed using Image J software (US National Institutes of Health, Bethesda, MD, USA).

### Fibrin gel bead assay

Fibrin gel bead assay can be used to model the entire process of angiogenesis [17], by seeding endothelial cells on the surface of Cytodex microcarrier beads followed by culture on the fibrin gel to form vessel structures. Briefly, 2500 pre-sterilized Cytodex® 3 microcarrier beads (C3275, Sigma-Aldrich) were mixed with 1 × 10^6^ HUVECs (p3 to 5) in 1.5 ml of EGM-2 medium (Lonza) within a FACS tube. The FACS tube was then placed within the incubator vertically with slight agitation every 20 minutes for more than 4 hours. Subsequently, 5 ml of fresh pre-warmed EGM-2 medium was added to the FACS tube, followed by pipetting to a T-25 flask for incubation at 37 °C and 5% CO_2_ overnight. Aliquots of 1 × 10^4^ cells (group 1: SHED-derived SMCs; group 2: SHED; group 3: primary SMCs) were mixed with 250 pre-coated beads in 500 μl of 2 mg/ml fibrinogen solution per well of a 24-well plate. The beads-fibrinogen solution was coagulated by adding thrombin (T7513, Sigma-Aldrich) at a concentration of 0.625 units/ml, and was then allowed to clot for 5 minutes at room temperature, followed by another 25 minutes incubation at 37 °C and 5% CO_2_. A total of 350 μl of fresh pre-warmed EGM-2 medium was then added to the surface of the clotted fibrin gel with medium being refreshed every 2 days. After 10 days, images were captured and analyzed using Image J software.

Subsequently, proteins of each group were extracted for further analysis. Briefly, gels were washed with cold PBS, and 250 μl of RIPA lysis buffer was added to each well, followed by transfer to a 1.5 ml Eppendorf tube. The gels and RIPA mixtures were then homogenized with two cycles of 30-second sonication and 20-second vortexing. The lysates were then centrifuged for 10 minutes at 4 °C, and the supernatant was collected for further analysis.

### Functional contraction study

The contractility assay was performed according to an established protocol [18]. Briefly, cells were detached using Trypsin, and the concentration was adjusted to 1.5 × 10^5^cells/ml. Then 0.4 ml of cell suspension and 0.2 ml of 3 mg/ml collagen solution (R&D Systems Minneapolis, MN, USA) were mixed thoroughly in a 1.6 ml Eppendorf tube (this volume is only for one well of a 24-well plate). After adding an appropriate volume of 1 M NaOH to the mixture of cells and collagen, 500 μl of the mixture was immediately transferred to a well of a 24-well plate. Gels were allowed to solidify for 20 minutes at room temperature, and then transferred into a 37 °C incubator with a humidified 5% CO_2_ atmosphere. The extent of gel contraction was measured by calculating the area of gel with the Image J software.

### Statistical analysis

All numerical data were expressed as mean ± standard deviation (SD). One-way ANOVA was utilized for statistical analysis. The threshold of statistical significance was set at *p* < 0.05.

## Results

### TGF-β1 can induce the differentiation of SHED into SMCs

**Fig. 1:**
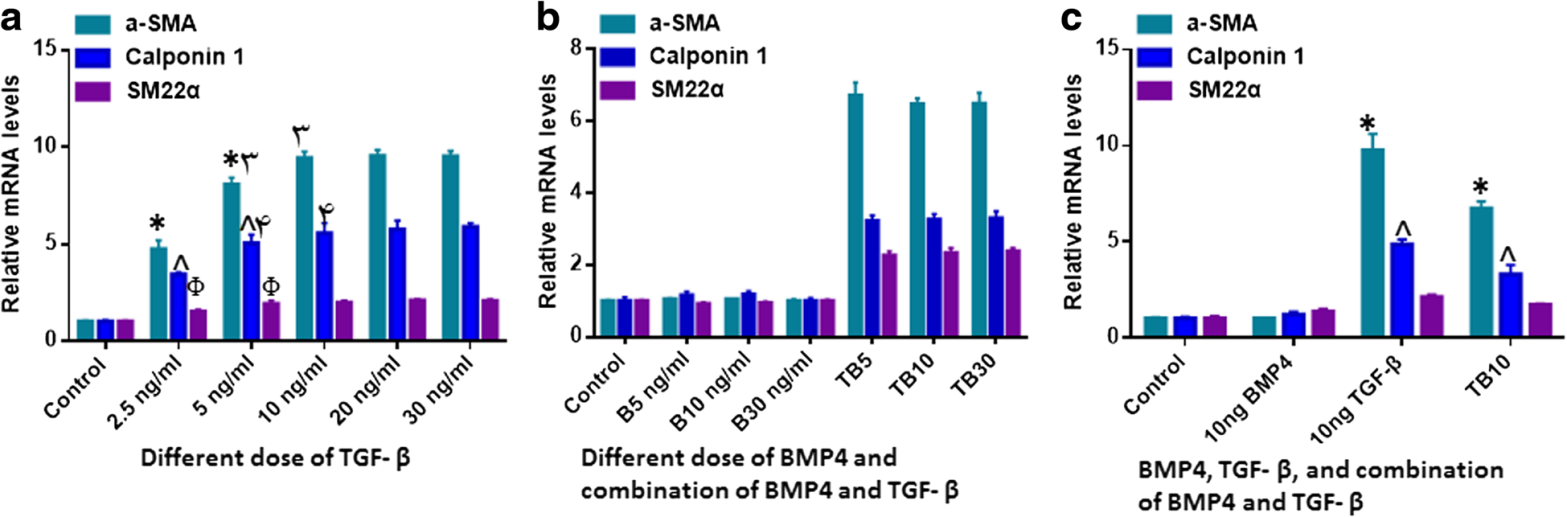
SMC-specific marker gene expression profile of SHED-derived SMCs induced with different concentrations and combinations of TGF-β1 and BMP4. Expression levels of SMC-specific marker genes relative to GAPDH with (**a**) different doses of TGF-β1 induction. There was significant upregulation of α-SMA and Calponin 1 gene expression levels as the TGF-β1 concentration is increased from 2.5 ng/ml to 10 ng/ml (*p* < 0.05). There were however no significant differences between the 10 ng/ml, 20 ng/ml and 30 ng/ml TGF-β1 treatment groups (*p* > 0.05). SM22α gene expression was a little different, by which 5 ng/ml TGF-β1 had a same effect with 10 ng/ml, 20 ng/ml and 30 ng/ml. **b** Different doses of BMP4 induction and with different combinations of TGF-β (10 ng/ml) and BMP4 (5, 10, 30 ng/ml). There were no significant differences amongst the different doses of BMP4 (*p* > 0.05), and also no significant differences amongst the different combinations of TGF-β and BMP4 (*p* > 0.05). **c** 10 ng/ml TGF-β1, 10 ng/ml BMP4 and the combination of 10 ng/ml TGF-β1 with BMP4. The effect of TGF-β1 was weakened when combined with BMP4 (*p* < 0.05). * ^ Φ: *p* < 0.05. *B* means BMP4; *TB* means TGF-β and BMP4. All experiments were performed three times (*N* = 3). *BMP4* bone morphogenetic protein 4, *TGF-β1* transforming growth factor beta 1, *α-SMA* alpha-smooth muscle actin

As two distinct cytokines of the TGF-β family, TGF-β1 and BMP4 were arbitrarily selected to induce SMCs differentiation. To evaluate whether TGF-β1 and BMP4 could induce SHED to functional SMCs, various combinations and concentrations of TGF-β1 and BMP4 were investigated. As shown in Fig. 1a, after 7 days induction, TGF-β1 upregulated the mRNA expression levels of SMCs specific markers (α-SMA, SM22α and Calponin), but there was no significant differences between the different concentrations of 10 ng/ml, 20 ng/ml and 30 ng/ml. BMP4 alone exerted negligible effects on the differentiation of SHED into SMCs. However when it was used in combination with TGF-β1, the effect of TGF-β1 was weakened (Fig. 1b, c). Hence, only the dose of 10 ng/ml TGF-β1 was utilized in further studies.

**Fig. 2:**
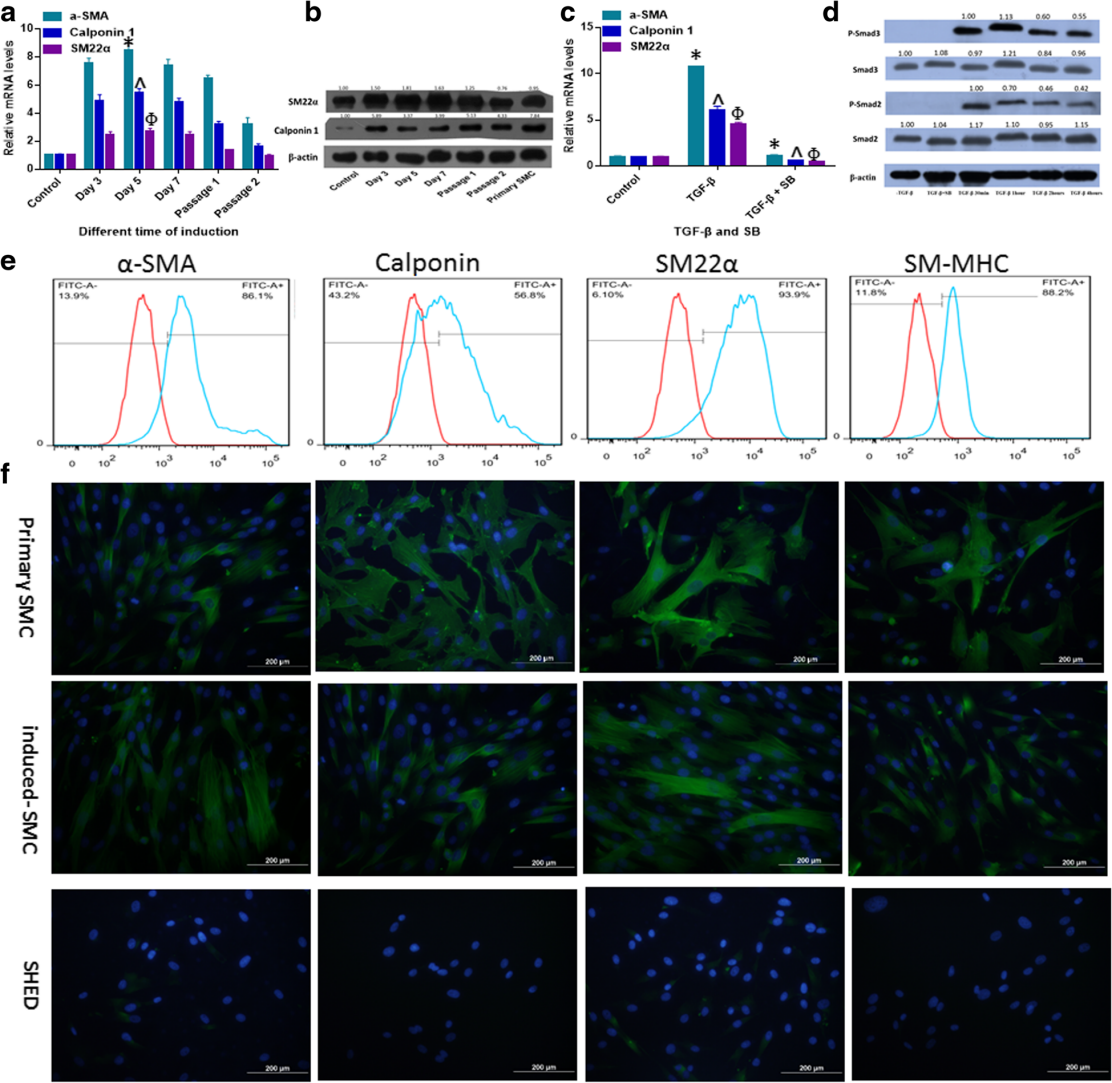
Characterization of SHED-derived SMCs. **a** Expression levels of SMC-specific marker gene expression relative to GAPDH at different induction time points. The expression levels of all SMC-specific marker genes peaked on day 5 (*p* < 0.05, compared with other time points). At passage 2, the gene expression levels of α-SMA and Calponin 1 declined but were still significantly higher than the control group (*p* < 0.05); while the gene expression level of SM22α declined to the same level as the control group (*p* > 0.05). * ^ Φ: *p* < 0.05 compared to all other time points. **b** The protein expression levels of SM22α and Calponin 1 were analyzed by western blot utilizing β-actin as the internal marker. Numbers depict the band density normalized against untreated controls and β-actin. **c** SB-431542 suppressed TGF-β1-mediated SMC differentiation. * ^ Φ: *p* < 0.05 between TGF-β1 group and SB-431542 + TGF-β1-treated group. **d** Smad2/3 became phosphorylated within 30 minutes of exposure to TGF-β1, and SB-431542 inhibited this phosphorylation. Numbers depict the band density normalized against untreated controls and β-actin. **e** SHED-derived SMCs were also analyzed for expression of SMC-specific markers (α-SMA, Calponin, SM22α and SM-MHC) with flow cytometry (α-SMA+ 86.1%, SM22α + 93.9%, Calponin + 56.8%, and SM-MHC+ 88.2% respectively) (the isotype control in *red*) and (**f**) immunofluorescence staining (nuclei in *blue*). All experiments were performed three times (*N* = 3). *SM-MHC* smooth muscle-myosin heavy chain, *SHED* stem cells from human exfoliated deciduous teeth, *α-SMA* alpha-smooth muscle actin

To assess the time-course effect of 10 ng/ml TGF-β1 on SMCs differentiation, we analyzed the mRNA (Fig. 2a) and protein (Fig. 2b) expression levels of SMCs specific markers on day 3, 5, and 7, passage1 and 2.The results showed that the expression of SMCs specific markers at the transcriptional level reached the highest level on day 5, with a slight decline on day 7. Although the expression levels of SMCs specific markers declined significantly by passage 1 and 2, these were still higher than undifferentiated SHED (except SM22α). As shown in Fig. 2c, SB-431542, a specific inhibitor of ALK5, could suppress TGF-β1-mediated SMC differentiation. To evaluate the signaling mechanisms involved in SHED-SMCs differentiation, we treated cells with TGF-β1 and evaluated the phosphorylation of Smad2/3. The results showed that Smad2/3 became phosphorylated within 30 minutes of exposure to TGF-β1, and that SB-431542 inhibited this phosphorylation (Fig. 2d).

To evaluate the efficiency of SMCs differentiation, the percentages of α-SMA, SM22α, Calponin, and smooth muscle-myosin heavy chain (SM-MHC) positive cells were quantified by flow cytometry analysis on day 7. As shown in Fig. 2e, high proportions of the cell population were positively expressing the various SMCs markers on day 7 (α-SMA^+^ 86.1%, SM22α^+^ 93.9%, Calponin^+^ 56.8%, and SM-MHC^+^ 88.2% respectively). Additionally, immunocytochemistry was also performed on day 7 (Fig. 2f).

### SHED-derived SMCs exhibit similar functional properties as primary SMCs

**Fig. 3:**
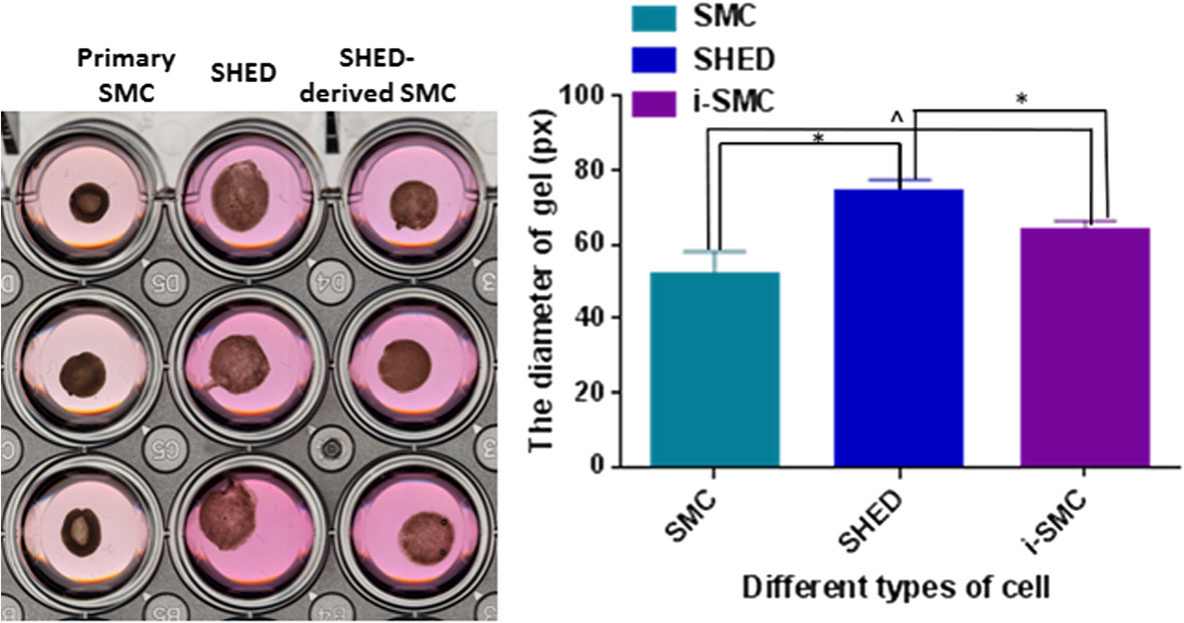
Collagen gel contraction assay. Gel surface areas were measured and further analyzed using the Image J software. *: *p* < 0.05, ^: *p* > 0.05. *HUVECs* human umbilical vein endothelial cells, *SHED* stem cells from human exfoliated deciduous teeth

**Fig. 4:**
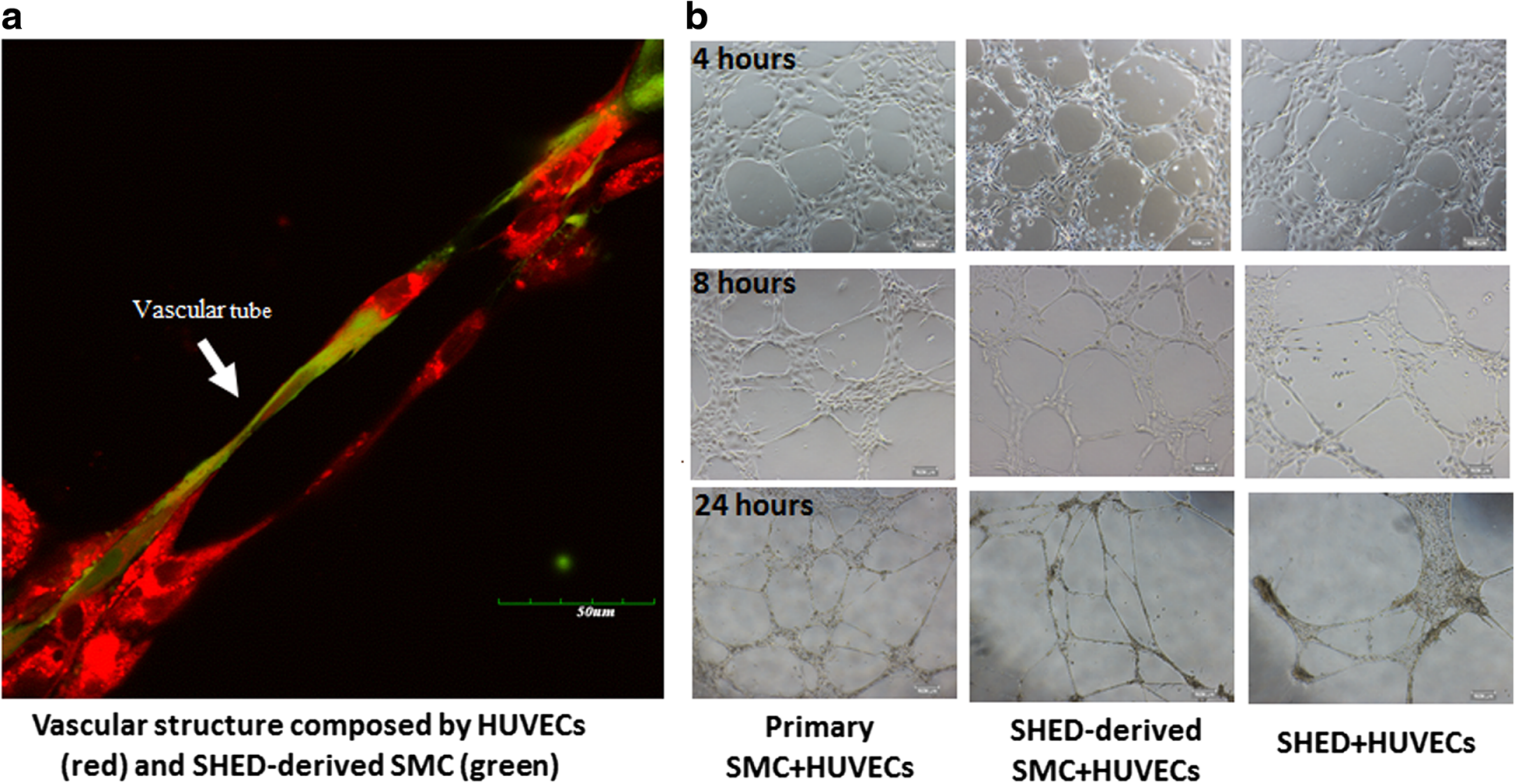
Vascular tube formation by SHED-derived SMCs and ECs on Matrigel. **a** Immunofluorescence image of vascular structure (EC in *red*; SHED-derived SMCs in *green*). (**b**) Phase-contrast images (×10) of vascular structures from 4 hours to 24 hours after seeding ECs and SHED-derived SMCs on Matrigel. *HUVECs* human umbilical vein endothelial cells, *SHED* stem cells from human exfoliated deciduous teeth, *SMCs* smooth muscle cells

The results of the contractility assay revealed that SHED-derived SMCs displayed similar contractile function as primary SMCs (Fig. 3). The results of the Matrigel tube formation assay showed that SHED-derived SMCs display close localization with endothelial cells. As shown in Fig. 4a, the co-culture of SHED-derived SMCs and HUVECs resulted in the formation of three-dimensional vessel structures that appear to recapitulate the interactions of ECs with SMCs in vivo. Furthermore, the time course of vascular structure formation by SHED-derived SMCs and HUVECs was similar to that generated by primary SMCs and HUVECs (Fig. 4b).

**Fig. 5:**
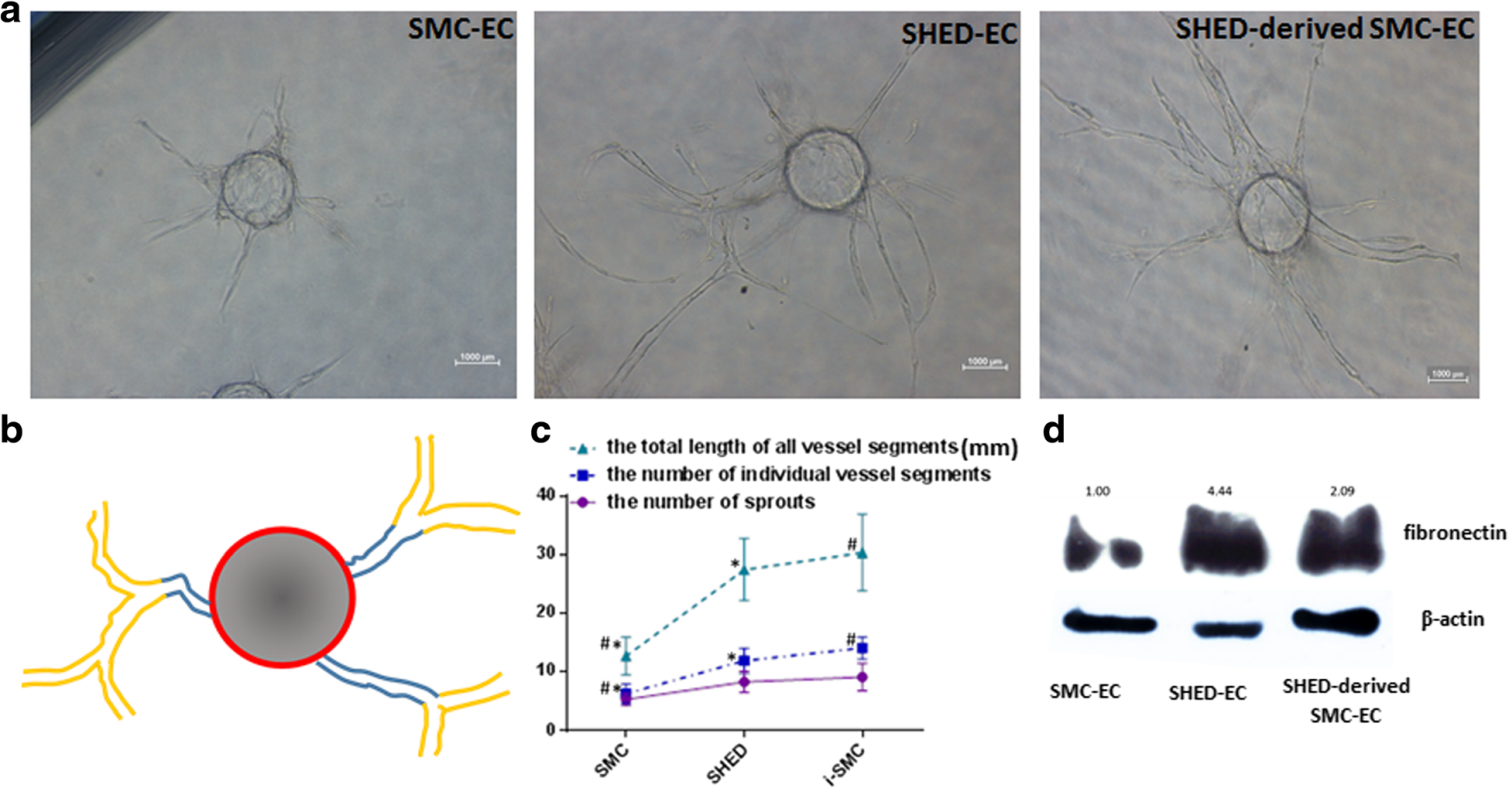
SHED-derived SMCs promote EC vessel formation. **a** Photomicrographs of EC-coated microbeads cultured in fibrin gels with SMC, SHED and SHED-induced SMC on the gel surface. **b** The schematic representation of calculating tube formation from each bead: (i) the number of vessels which sprout from the bead directly (in *blue*); (ii) the total number of individual vessel segments (including *blue* and *yellow*); and (iii) the total length of all the vessel segments. **c** Quantification of the vessel structures in the fibrin gel bead assay in the presence of SMC, SHED and SHED-induced SMC.*: *p* < 0.05 versus SMCs group, ^: *p* < 0.05 between SHED and SHED-derived SMCs. **d** Western blot showing fibronectin expression within each experimental group. All experiments were performed three times (*N* = 3). *ECs* endothelial cells, SHED stem cells from human exfoliated deciduous teeth, SMCs smooth muscle cells

The three-dimensional fibrin gel bead assay demonstrated the capacity of SHED-derived SMCs to promote tube formation. Figure 5a provides a schematic representation of calculating tube formation from fibrin beads [19]. The results showed that SHED-derived SMCs had a stronger capacity than primary SMCs in promoting tube formation (Fig. 5b, c).

To further investigate the mechanisms by which the formation of vessels is regulated by perivascular cells, the protein expression levels of Fibronectin were assessed. The results showed that cultures containing SHED-derived SMCs exhibited higher expression levels of Fibronectin than the primary SMCs group (Fig. 5d).

## Discussion

Primary human SMCs obtained from either autologous or allogeneic sources are not an ideal candidate for vascular tissue engineering due to their low replicative capacity and scarce availability [20]. Human pluripotent stem cells [5, 12], human induced pluripotent stem cells [21], and bone marrow-derived mesenchymal stem cells [22] have been demonstrated to possess the capacity to generate functional SMCs, but there are still some obstacles limiting their clinical applications, such as ethical concerns pertaining to the cell sources [23], as well as the risk of carcinogenesis after transplantation [24].

As a novel subpopulation of adult stem cells, stem cells from human exfoliated deciduous teeth (SHED), which are derived from a naturally occurring exfoliated tissue, present a unique stem cell source for potential clinical applications [25]. Besides their multi-potential differentiation capacity to differentiate into the neural, osteoblast, and adipocyte lineages [26, 27], SHED have also been demonstrated recently to differentiate into endothelial cells, which involves the MEK1/ERK signaling pathway [8]. However, there is as yet no report of SHED differentiating into SMCs. In this study, we demonstrated that under the stimulatory effects of TGF-β1, SHED could differentiate into functional SMCs. α-SMA is the most widely recognized marker of SMCs [28], but it is still necessary to combine this with other markers to positively identify SMCs. These include SM22α, Calponin, and particularly SM-MHC, which are believed to be expressed only in contractile SMCs [29]. The results of this study showed that all these SMCs markers could be detected (like Calponin) or enhanced (like α-SMA) when SHED were stimulated with TGF-β1. Furthermore, upon expanding the SHED-derived SMCs to passage 1 and 2, there was observed to be stable expression of α-SMA, SM22α, Calponin, and SM-MHC at passage 1.

Besides exhibiting the typical phenotype of SMCs, mature vascular SMCs also need to display functional characteristics, such as the ability to stabilize blood vessels, promote vascular formation and display contractile function to regulate blood pressure [2]. During the course of in vivo vascular formation, primary SMCs are recruited to the vicinity of vessels formed by endothelial cells through extracellular matrix (ECM) production and cytokine interaction, and serve to stabilize the nascent capillary during the early stages of vessel formation [30]. At later stages, these SMCs acquire a contractile function under the effects of different stimulatory cues [31].

To evaluate the ability of SHED-derived SMCs in stabilizing and promoting engineered vascular networks, two three-dimensional angiogenesis assays were performed in this study. First, we examined the in vitro formation of capillary-like structures by co-culture of HUVECs and SHED-derived SMCs on Matrigel. Because the in vitro Matrigel angiogenesis assay is rapid, reliable and quantitative, it has been widely used to study the regulatory mechanisms of angiogenesis [32]. The observed long-term stability would thus indicate that SHED-derived SMCs had similar capacity to generate vascular-like structures as primary SMCs. Second, we examined the capacity of perivascular cells to enhance the process of vascularization in fibrin gel. The number of vessels which sprout from the beads directly, the total number of individual vessel segments and the total length of all the vessel segments were three parameters utilized in evaluating vascular structures in the fibrin gel bead assay [20]. It is interesting that the capacity of SHED-derived SMCs to enhance vascular formation was better than primary SMCs. ECM proteins, particularly fibronectin, have been widely reported as inductive factors in regulating angiogenesis. An in vivo study showed that fibronectin-binding integrin α5β1 can enhance angiogenesis [33], and this result was further confirmed by another study [34]. Therefore, we attempted to analyze fibronectin expression. The results showed that higher expression levels of fibronectin were detected in the vessel structures composed of HUVECs and SHED-derived SMCs, as compared to the HUVECs with primary SMCs group. These findings led us to propose a hypothesis that stem cells with strong capacity for producing ECM make them better at promoting angiogenesis than SMCs.

Contractility is a key characteristic and distinguishing property of mature SMCs, which enables blood vessels to control appropriate blood pressure through alteration of the luminal diameter by contraction and relaxation [18]. An in vitro assay utilizing collagen gel for monitoring the contraction of smooth muscle cells has been widely accepted as a classical test model for assaying contractility [18]. In this study, SHED-derived SMCs were found to be capable of contracting when cultured in collagen gel, thus exhibiting similar functional characteristics as primary SMCs.

As a superfamily of growth factors and cytokines, the TGF-β superfamily includes the various TGF-β isoforms, Nodals, Activin, and BMPs, which play crucial roles in diverse biological processes, such as cell differentiation [35]. The initiation of TGF-β signaling is triggered through the binding of TGF-β cytokines to their cognate receptors (TGF-β RI and RII). Upon ligand binding, the activated TGF-β RI recruits and phosphorylates Smad proteins, i.e. Smad 2/3 for TGF-βs, Activin, Nodals, and Smad 1/5/8 for BMPs [35]. Many studies [36, 37] have reported that it is Smad 2/3 which play an important role in SMCs differentiation. However, there are also studies [13, 38] which have shown that BMP4 could mediate SMCs differentiation via the Smad 1/5/8 pathway. Therefore in our study, we selected TGF- β1 and BMP4 as two representative growth factors of the TGF-β superfamily to induce SMCs differentiation. The results of this study conclusively demonstrated that BMP4 alone exerted negligible effects on the differentiation of SHED into SMCs. Furthermore, the effects of TGF-β1 on SHED-SMCs differentiation could be weakened when combined with BMP4. We hypothesize that BMP4 might compete with TGF-β1 for binding to TGF-β receptors (TGF-β RI and RII) during the process of SHED-SMCs differentiation. Although accumulated evidence shows that BMPs play crucial roles in the regulation of stem cell properties and lineage fate, their functions vary with different stem cell types. While the differentiation of SHED into SMCs could be mediated by TGF-β1, no significant differences were observed between concentrations of 10 ng/ml to 30 ng/ml TGF-β1. The results of our analysis of the Smad signaling pathway showed that upon interaction with TGF-β1, the TGF receptors recruited and phosphorylated the downstream targets of Smad 2 and 3. Besides this classical signaling pathway, several other signaling pathways that affect SMC differentiation under TGF-β1 stimulation have also been reported, such as the p38 [39], AKT [40], and RhoA [41] signaling pathways. In this study, we investigated the effects of SB-431542, a specific inhibitor of ALK5 [42], on the differentiation of SHED into SMCs. In the presence of SB-431542, SHED could not be induced into SMCs under TGF-β1 stimulation, which thus confirmed the crucial role of the TGF-β1-ALK5 signaling pathway in modulating the differentiation of SHED into SMCs. This finding could explain why BMP4 had negligible effects on the course of differentiation of SHED into SMCs. Unlike TGF-β1, BMP4 functions through Alk2, Alk3 and Alk6 that mediate phosphorylation of Smad1, Smad5, or Smad8, which in turn modulate target gene expression at the transcriptional level [43].

## Conclusions

Our data confirmed that SHED possess the capacity to differentiate into functional smooth muscle cells. Further analysis of the results revealed that TGF-β1 regulate SHED differentiation into smooth muscle via the ALK5 signaling pathway. Hence, SHED appear to be a promising candidate cell type for vascular tissue engineering.

## Additional files

Additional file 1: Figure S1. The flow cytometry results of expression of stem cell-associated phenotypic markers, CD105, CD90, CD73, and CD45. (TIF 249 kb)
Additional file 2: Figure S2. The multiple differentiation capacity of SHED: (A) osteogenic, (B) neurogenic, and (C) adipogenic. (TIF 1885 kb)
Additional file 3: Figure S3. The statistical analysis of the western blot results: (A) the protein expression levels of SM22α and Calponin 1 in different time points; * #: *p* < 0.05 versus control group; (B) the phosphorylation and total protein of Smad2/3; *: *p* < 0.05 versus TGF-β 30-minute group; * #: *p* < 0.05 versus TGF-β group; (B) the fibronectin expression; *: *p* < 0.05 versus SMC-EC group. (TIF 343 kb)

## Abbreviations

BMP4: bone morphogenetic protein 4
ECM: extracellular matrix
hESCs: human embryonic stem cells
HUVECs: human umbilical vein endothelial cells
IgG: immunoglobulin G
RT-qPCR: quantitative real-time polymerase chain reaction
SHED: stem cells from human exfoliated deciduous teeth
SM-MHC: smooth muscle-myosin heavy chain
TGF-β1: transforming growth factor beta 1
vSMCs: vascular smooth muscle cells
α-SMA: alpha-smooth muscle actin

## References

[1] Adams RH, Alitalo K (2007). Molecular regulation of angiogenesis and lymphangiogenesis. Nat Rev Mol Cell Biol.

[2] Carmeliet P (2000). Mechanisms of angiogenesis and arteriogenesis. Nat Med.

[3] Gökçinar-Yagci B, Uçkan-Çetinkaya D, Çelebi-Saltik B (2015). Pericytes: properties, functions and applications in tissue engineering. Stem Cell Rev.

[4] Kim S, von Recum H (2008). Endothelial stem cells and precursors for tissue engineering: cell source, differentiation, selection, and application. Tissue Eng Part B Rev.

[5] Patsch C, Challet-Meylan L, Thoma EC, Urich E, Heckel T, O'Sullivan JF (2015). Generation of vascular endothelial and smooth muscle cells from human pluripotent stem cells. Nat Cell Biol.

[6] Oyamada N, Itoh H, Sone M, Yamahara K, Miyashita K, Park K (2008). Transplantation of vascular cells derived from human embryonic stem cells contributes to vascular regeneration after stroke in mice. J Transl Med.

[7] Nakamura S, Yamada Y, Katagiri W, Sugito T, Ito K, Ueda M (2009). Stem cell proliferation pathways comparison between human exfoliated deciduous teeth and dental pulp stem cells by gene expression profile from promising dental pulp. J Endod.

[8] Bento LW, Zhang Z, Imai A, Nör F, Dong Z, Shi S, Araujo FB, Nör JE (2013). Endothelial differentiation of SHED requires MEK1/ERK signaling. J Dent Res.

[9] Zhang Z, Nor F, Oh M, Cucco C, Shi S, Nör JE (2016). Wnt/β-catenin signaling determines the vasculogenic fate of post-natal mesenchymal stem cells. Stem Cells.

[10] Shi N, Xie WB, Chen SY (2012). Cell division cycle 7 is a novel regulator of transforming growth factor-β-induced smooth muscle cell differentiation. J Biol Chem.

[11] Sinha S, Wamhoff BR, Hoofnagle MH, Thomas J, Neppl RL, Deering T (2006). Assessment of contractility of purified smooth muscle cells derived from embryonic stem cells. Stem Cells.

[12] Vo E, Hanjaya-Putra D, Zha Y, Kusuma S, Gerecht S (2010). Smooth-muscle-like cells derived from human embryonic stem cells support and augment cord-like structures in vitro. Stem Cell Rev.

[13] Wang C, Yin S, Cen L, Liu Q, Liu W, Cao Y (2010). Differentiation of adipose-derived stem cells into contractile smooth muscle cells induced by transforming growth factor-beta1 and bone morphogenetic protein-4. Tissue Eng Part A.

[14] Narita Y, Yamawaki A, Kagami H, Ueda M, Ueda Y (2008). Effects of transforming growth factor-beta 1 and ascorbic acid on differentiation of human bone-marrow-derived mesenchymal stem cells into smooth muscle cell lineage. Cell Tissue Res.

[15] Yuan C, Wang P, Zhu L, Dissanayaka WL, Green DW, Tong EH (2015). Coculture of stem cells from apical papilla and human umbilical vein endothelial cell under hypoxia increases the formation of three-dimensional vessel-like structures *in vitro*. Tissue Eng Part A.

[16] Arnaoutova I, Kleinman HK (2010). In vitro angiogenesis: endothelial cell tube formation on gelled basement membrane extract. Nat Protoc.

[17] Conway EM, Collen D, Carmeliet P (2001). Molecular mechanisms of blood vessel growth. Cardiovasc Res.

[18] Ngo P, Ramalingam P, Phillips JA, Furuta GT (2006). Collagen gel contraction assay. Methods Mol Biol.

[19] Griffith CK, Miller C, Sainson RC, Calvert JW, Jeon NL, Hughes CC, George SC (2005). Diffusion limits of an in vitro thick prevascularized tissue. Tissue Eng.

[20] Poh M, Boyer M, Solan A, Dahl SL, Pedrotty D, Banik SS (2005). Blood vessels engineered from human cells. Lancet.

[21] Bajpai VK, Mistriotis P, Loh YH, Daley GQ, Andreadis ST (2012). Functional vascular smooth muscle cells derived from human induced pluripotent stem cells via mesenchymal stem cell intermediates. Cardiovasc Res.

[22] Gong Z, Niklason LE (2008). Small diameter human vessel wall engineered from bone marrow-derived mesenchymal stem cells (hMSCs). FASEB J.

[23] Takahashi K, Yamanaka S (2006). Induction of pluripotent stem cells from mouse embryonic and adult fibroblast cultures by defined factors. Cell.

[24] Gore A, Li Z, Fung HL, Young JE, Agarwal S, Antosiewicz-Bourget J (2011). Somatic coding mutations in human induced pluripotent stem cells. Nature.

[25] Cordeiro MM, Dong Z, Kaneko T, Zhang Z, Miyazawa M, Shi S (2008). Dental pulp tissue engineering with stem cells from exfoliated deciduous teeth. J Endod.

[26] Nourbakhsh N, Soleimani M, Taghipour Z, Karbalaie K, Mousavi SB, Talebi A (2011). Induced in vitro differentiation of neural-like cells from human exfoliated deciduous teeth-derived stem cells. Int J Dev Biol.

[27] Leone A, Volponi AA, Renton T, Sharpe PT (2012). In-vitro regulation of odontogenic gene expression in human embryonic tooth cells and SHED cells. Cell Tissue Res.

[28] Owens GK, Kumar MS, Wamhoff BR (2004). Molecular regulation of vascular smooth muscle cell differentiation in development and disease. Physiol Rev.

[29] Rensen SS, Doevendans PA, van Eys GJ (2007). Regulation and characteristics of vascular smooth muscle cell phenotypic diversity. Neth Heart J.

[30] Jain RK (2003). Molecular regulation of vessel maturation. Nat Med.

[31] Tsai MC, Chen L, Zhou J, Tang Z, Hsu TF, Wang Y (2009). Shear stress induces synthetic-to-contractile phenotypic modulation in smooth muscle cells via peroxisome proliferator-activated receptor alpha/delta activations by prostacyclin released by sheared endothelial cells. Circ Res.

[32] Arnaoutova I, George J, Kleinman HK, Benton G (2009). The endothelial cell tube formation assay on basement membrane turns 20: state of the science and the art. Angiogenesis.

[33] Kim S, Bell K, Mousa SA, Varner JA (2000). Regulation of angiogenesis in vivo by ligation of integrin alpha5beta1 with the central cell-binding domain of fibronectin. Am J Pathol.

[34] Newman AC, Chou W, Welch-Reardon KM, Fong AH, Popson SA, Phan DT (2013). Analysis of stromal cell secretomes reveals a critical role for stromal cell-derived hepatocyte growth factor and fibronectin in angiogenesis. Arterioscler Thromb Vasc Biol.

[35] Derynck R, Zhang YE (2003). Smad-dependent and Smad-independent pathways in TGF-β family signaling. Nature.

[36] Chen S, Lechleider RJ (2004). Transforming growth factor-beta-induced differentiation of smooth muscle from a neural crest stem cell line. Circ Res.

[37] Kalinina N, Agrotis A, Antropova Y, Ilyinskaya O, Smirnov V, Tararak E, Bobik A (2004). Smad expression in human atherosclerotic lesions: evidence for impaired TGF-beta/Smad signaling in smooth muscle cells of fibrofatty lesions. Arterioscler Thromb Vasc Biol.

[38] Yu PB, Deng DY, Beppu H, Hong CC, Lai C, Hoyng SA, Kawai N, Bloch KD (2008). Bone morphogenetic protein (BMP) type II receptor is required for BMP-mediated growth arrest and differentiation in pulmonary artery smooth muscle cells. J Biol Chem.

[39] Deaton RA, Su C, Valencia TG, Grant SR (2005). Transforming growth factor-β1-induced expression of smooth muscle marker genes involves activation of PKN and p38 MAPK. J Biol Chem.

[40] Lien SC, Usami S, Chien S, Chiu JJ (2006). Phosphatidylinositol 3-kinase/Akt pathway is involved in transforming growth factor-β1-induced phenotypic modulation of 10 T1/2 cells to smooth muscle cells. Cell Signal.

[41] Chen S, Crawford M, Day RM, Briones VR, Leader JE, Jose PA (2006). RhoA modulates Smad signaling during transforming growth factor-β-induced smooth muscle differentiation. J Biol Chem.

[42] Inman GJ, Nicolás FJ, Callahan JF, Harling JD, Gaster LM, Reith AD (2002). SB-431542 is a potent and specific inhibitor of transforming growth factor-superfamily type I Activin Receptor-Like Kinase (ALK) Receptors ALK4, ALK5, and ALK7. Mol Pharma Col.

[43] Zhang J, Li L (2005). BMP signaling and stem cell regulation. Dev Biol.

