# Supplementary figures and images for: TGF-β1-induced differentiation of SHED into functional smooth muscle cells
Source: Stem Cell Res Ther. 2017 Jan 23;8:10. doi: 10.1186/s13287-016-0459-0 (PMC5260045; doi:10.1186/s13287-016-0459-0)

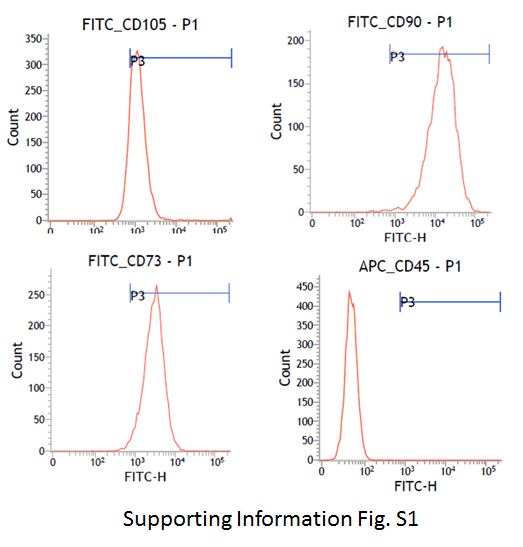

Supplement: Additional file 1: Figure S1. — The flow cytometry results of expression of stem cell-associated phenotypic markers, CD105, CD90, CD73, and CD45. (TIF 249 kb) [file 13287_2016_459_MOESM1_ESM.tif]

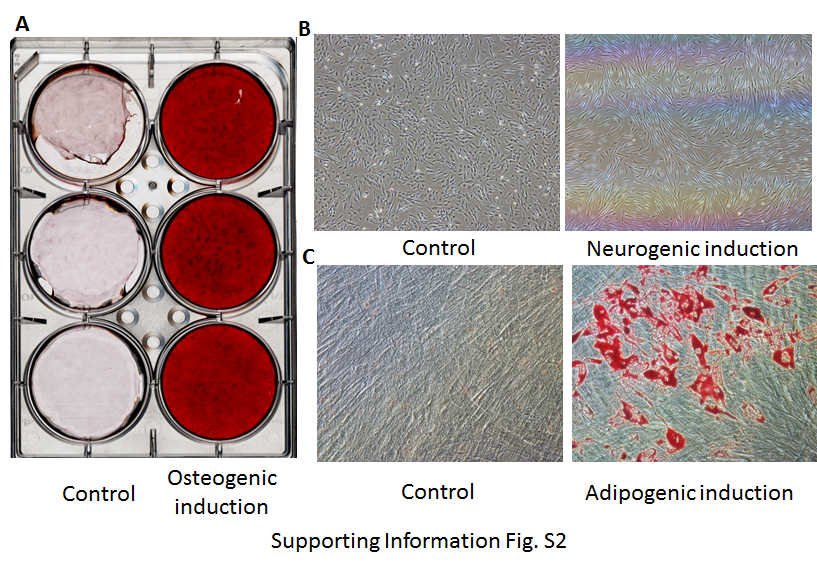

Supplement: Additional file 2: Figure S2. — The multiple differentiation capacity of SHED: (A) osteogenic, (B) neurogenic, and (C) adipogenic. (TIF 1885 kb) [file 13287_2016_459_MOESM2_ESM.tif]

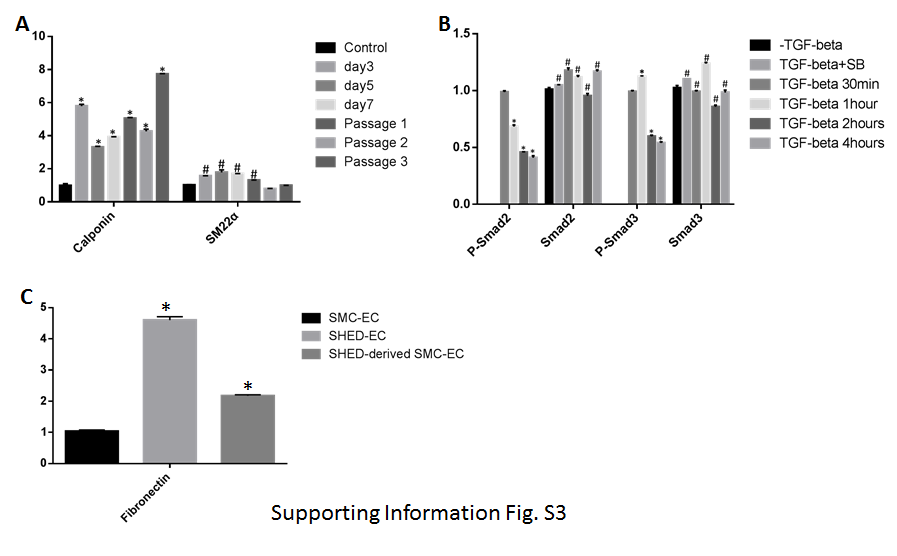

Supplement: Additional file 3: Figure S3. — The statistical analysis of the western blot results: (A) the protein expression levels of SM22α and Calponin 1 in different time points; * #: p < 0.05 versus control group; (B) the phosphorylation and total protein of Smad2/3; *: p < 0.05 versus TGF-β 30-minute group; * #: p < 0.05 versus TGF-β group; (B) the fibronectin expression; *: p < 0.05 versus SMC-EC group. (TIF 343 kb) [file 13287_2016_459_MOESM3_ESM.tif]
